# Permeation Properties of Ions through Inorganic Silica-Based Membranes

**DOI:** 10.3390/membranes10020027

**Published:** 2020-02-08

**Authors:** Junko Yoshiura, Katsunori Ishii, Yuta Saito, Takaya Nagataki, Yuhei Nagataki, Ayumi Ikeda, Mikihiro Nomura

**Affiliations:** 1 Department of Applied Chemistry, Shibaura Institute of Technology, 3-7-5 Toyosu, Koto-ku, Tokyo 135-8548, Japan; mc19025@shibaura-it.ac.jp (J.Y.); na19101@shibaura-it.ac.jp (K.I.); ad16043@shibaura-it.ac.jp (Y.S.); ad16074@shibaura-it.ac.jp (T.N.); ad16075@shibaura-it.ac.jp (Y.N.); 2 National Institute of Advanced Industrial Science and Technology (AIST), 1-1-1 Higashi, Tsukuba, Ibaraki 305-8565, Japan; a-ikeda@aist.go.jp

**Keywords:** silica membrane, counter diffusion CVD method, chemical vapor deposition, reverse osmosis, nanofiltration, ion separation, fluorine silica precursor

## Abstract

The development of inorganic membranes has mainly found applicability in liquid separation technologies. However, only a few reports cite the permeation and separation of liquids through inorganic nanofiltration membranes compared with the more popular microfiltration membranes. Herein, we prepared silica membranes using 3,3,3-trifluoropropyltrimethoxysilane (TFPrTMOS) to investigate its liquid permeance performance using four different ion solutions (i.e., NaCl, Na_2_SO_4_, MgCl_2_, and MgSO_4_). The TFPrTMOS-derived membranes were deposited above a temperature of 175 °C, where the deposition behavior of TFPrTMOS was dependent on the organic functional groups decomposition temperature. The highest membrane rejection was from NaCl at 91.0% when deposited at 200 °C. For anions, the SO_4_^2−^ rejections were the greatest. It was also possible to separate monovalent and divalent anions, as the negatively charged groups on the membrane surfaces retained pore sizes >1.48 nm. Ions were also easily separated by molecular sieving below a pore size of 0.50 nm. For the TFPrTMOS-derived membrane deposited at 175 °C, glucose showed 67% rejection, which was higher than that achieved through the propyltrimethoxysilane membrane. We infer that charge exclusion might be due to the dissociation of hydroxyl groups resulting from decomposition of organic groups. Pore size and organic functional group decomposition were found to be important for ion permeation.

## 1. Introduction

The technique of liquid separation using inorganic membranes has been one of the most compelling developments in recent decades, as they have the potential for highly selective separations through manipulation of their thermal and chemical properties [1,2]. In particular, sintered inorganic microfiltration (MF) membranes have had ample interest, especially for industrial applications. Here, we have focused on inorganic nanofiltration (NF) membranes and reverse osmosis (RO) membranes. For NF membranes, their permeation properties can be described simply through molecular sieving and charge effects. Compared with RO, modern NF membranes have been used successfully for the production of ultrapure water in semiconductor production facilities due to the higher fluxes they administer. For example, commercial NF membranes such as aromatic polyamides, polypiperazine amides, and sulfonated polyethersulfones, have all been favorably applied for water treatment. Yuan et al. [3] reported the ion permeation behavior of the polyamide membrane where the ion rejections through a 1,2,3,4-cyclobutanetetracarboxylic acid chloride membrane showed >94% for Ca^2+^, Mg^2+^, and SO_4_^2−^ with a water flux of about 120 kg m^−2^ h^−1^ at 3.0 MPa. This high rejection was said to be due to the molecular sieving effect.

Inorganic membranes, such as zeolites, have also been investigated for use in gas separations, vapor separations, and pervaporation [4,5,6,7,8,9]. However, only a few reports [9,10,11] on liquid permeation and separation through inorganic NF membranes have been published to date. Zhu et al. [9] reported on the ion separation performance of MF type zeolite membranes using Fe^3+^, Al^3+^, Mg^2+^, and SO_4_^2−^, with hydration diameters of 0.91, 0.95, 0.87, and 0.76 nm, respectively. The ion rejections for Fe^3+^, Al^3+^, Mg^2+^, and SO_4_^2−^ were all found to be over 80% due to charge interactions between the ions and the surface of the zeolite membrane. Tsuru et al. [11] reported on the NaCl permeation of sol–gel-derived silica membranes. These 1,2-bis(triethoxysilyl)ethane-derived membranes possessed a water flux of 1.5 × 10^−13^ m^3^ m^−2^ s^−1^ Pa^−1^ with the NaCl rejection reaching 98%. The flux was also at a similar level to that of the polymeric membrane SW30HR with a permeance of 1.1 × 10^−12^ m^3^ m^−2^ s^−1^ Pa^−1^.

**Figure 1:**
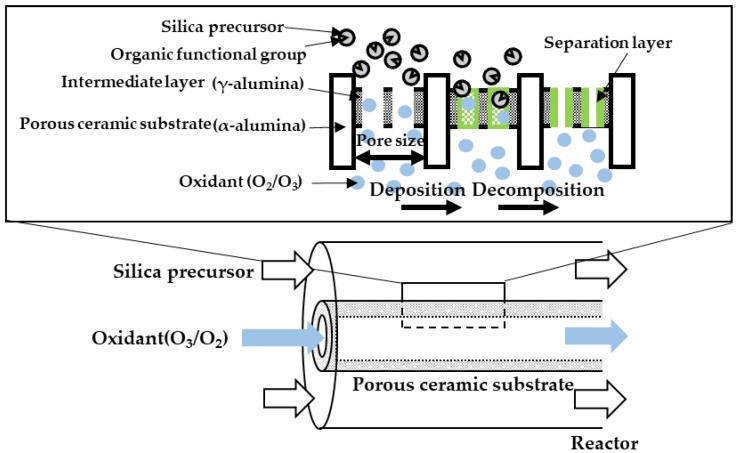
A schematic diagram of the counter diffusion chemical vapor deposition method.

Our research group has been developing silica membranes for some time using a counter diffusion chemical vapor deposition (CVD) method shown in Figure 1 [12,13,14,15,16,17,18,19,20,21]. Here, a silica precursor was used on one side of a porous ceramic substrate, with an oxidant supplied to the opposite side. Amorphous silica was then deposited into the pores of the substrate. The reaction ceased by depositing silica inside the pores to generate a uniformly thin silica surface layer. Ikeda et al. [22,23] investigated the effects of organic groups on these silica precursors using different pore sizes. The pore sizes were controlled precisely in angstroms (Å). The total flux was found to be 5.8 kg m^−2^ h^−1^ with a 92% rejection using a silica membrane derived from diphenyldimethoxysilane. The liquid permeation performances at 100 ppm H_2_SO_4_ were also achieved using this membrane, with the effects of charge on the rejection further discussed. On the basis of these studies, we see a potential for favorable ion separations using silica membranes derived from the CVD method, even if the effects of ion permeation are not clear.

Therefore, in this paper, the ion permeation behavior of silica membranes prepared by the counter diffusion CVD method were investigated, particularly tracking the effects of some unique hydrophobic membranes prepared from 3,3,3-trifluoropropyltrimethoxysilane (TFPrTMOS). We show the important effects of surface charge suppression on the molecular sieves of the membrane by performing liquid permeation tests with four kinds of ionic solutions (namely, NaCl, Na_2_SO_4_, MgCl_2_, and MgSO_4_).

## 2. Materials and Methods

### 2.1. Porous Ceramic Substrates

The γ-alumina substrates consisted of an α-alumina porous tube with a length of 9.5 cm, φ = 10 mm, and a pore size of 150 nm (Noritake Co., Nagoya, Japan) and an γ-alumina intermediate layer with a pore size of 4 nm. The γ-alumina intermediate layer was coated onto the α-alumina porous tube using a coating solution of 3.5 wt.% γ-alumina sol and a 3:5 vol/vol solution of polyvinyl alcohol at the center of a 3.0 cm membrane. Both ends of the substrate were sealed through glass coating.

### 2.2. Thermogravimetric Analysis of the Hydrolysis Silica Powder

The thermal decomposition behavior of the organic functional group in propyltrimethoxysilane (PrTMOS) and TFPrTMOS was examined by thermogravimetric (TG) analysis (TGA-50, Shimazu Co., Kyoto, Japan) using hydrolyzed silica powders. The hydrolyzed silica powders were prepared using a molar ratio of Si: NaOH:H_2_O:EtOH = 1:0.94:3.8:25. After stirring at 60 °C for 30 min, the silica sols were dried at 100 °C for conversion into hydrolyzed powders PrTMOS and TFPrTMOS (herein, referred to as PrTMOS powder and TFPrTMOS powder, respectively). The measurement was conducted at an O_3_ flow rate of 50 Ml min^−1^ and a heating rate of 3 °C min^−1^. The weight fraction was a differential function of the weight percentage measured by TG.

### 2.3. The Counter Diffusion CVD Method

**Figure 2:**
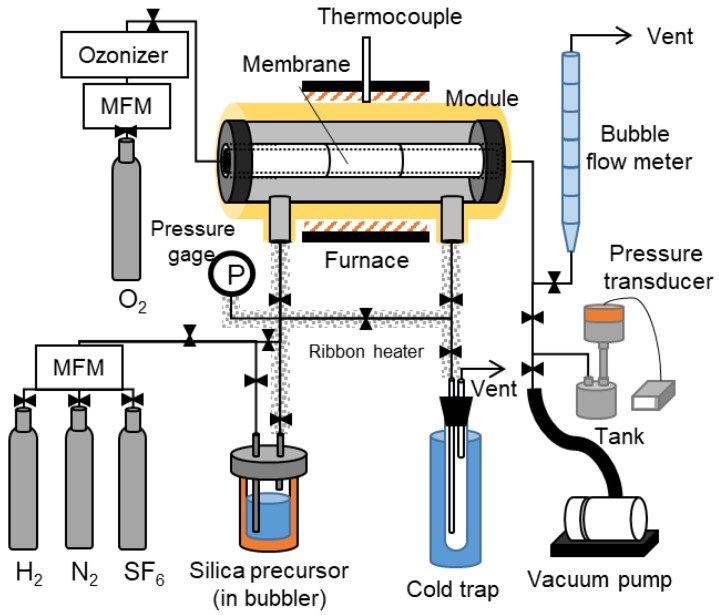
A schematic diagram of the counter diffusion CVD-gas permeation apparatus.

The silica membranes were prepared using the counter diffusion CVD method. Both PrTMOS and TFPrTMOS (Shin-Etsu Chem. Co., Tokyo, Japan) were used as the silica precursors. Figure 2 shows a schematic diagram of the apparatus used for the counter diffusion CVD. Here, the silica precursor was placed inside a bubbler and kept at a constant temperature of 75 °C. The precursor vapor was supplied to the outer side of the substrate at an N_2_ flow rate of 0.2 L min^−1^. Ozone was also produced by an ozone generator (ZOS-YB-20G, Shoken Co., Saitama, Japan) and introduced into the inner side of the substrate at a rate of 0.2 L min^−1^. The deposition was carried out between 100 °C and 270 °C for a period of 60 min.

### 2.4. Single Gas Permeation Tests

The single gas permeation tests were carried out for gases H_2_ (0.28 nm), N_2_ (0.36 nm), and SF_6_ (0.55 nm) each at ambient temperature. The gas permeance was calculated using Equation (1);

(1) $$P_{i}=\frac{n_{i}}{t\cdot A\cdot \Delta p}$$

where *n_i_* [mol] are the permeated molecules, *t* [s] is the permeation time, *A* [m^2^] is the membrane area, and Δ*p* [Pa] is the pressure difference.

### 2.5. Liquid Permeation Tests

**Figure 3:**
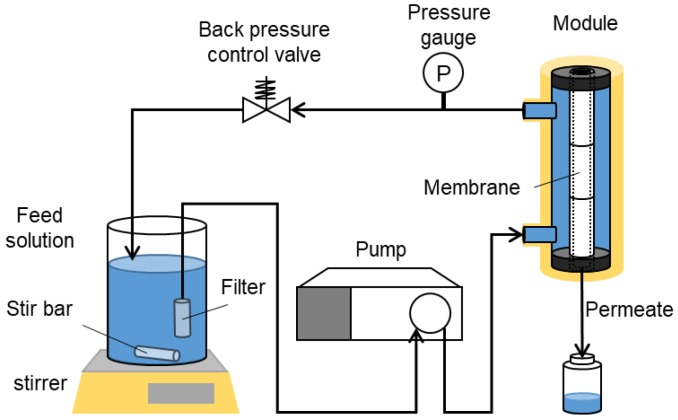
A diagram of the liquid permeation apparatus.

Figure 3 shows a schematic diagram of the apparatus used for the liquid permeation tests. Here, the liquid permeation performances were evaluated using glucose with a Stokes diameter of 0.72 nm, along with NaCl, Na_2_SO_4_, MgCl_2_, and MgSO_4_ (Table 1 and Table 2). Further details on the solutes used are summarized in Table 1. All solute concentrations in the feed were 100 ppm. The comparison using equal mass concentration was acceptable because the highest ration of salt molar concentrations is low (=ca. 1.5). The permeation tests were also carried out at ambient temperature using a feed pressure of 6.0 MPa.

The solute concentrations were measured using a TOC-L total organic carbon meter (Shimazu Co., Kyoto, Japan) and an ES-71 electrical conductivity meter (Horiba Advanced Techno Co., Japan). The flux (*J* [kg m^−2^ h^−1^]) and the rejection (*R* [%]) were each calculated using Equations (2) and (3):

(2) $$R=\left(1-\frac{C_{p}}{C_{b}}\right)\times 100$$

(3) $$J=\frac{W}{A\cdot t}$$

where *C_p_* and *C_b_* [mol L^−1^] are the concentrations of the permeate and feed solutions, respectively, and *W* [kg] is the total weight of the permeate solution.

## 3. Results and Discussion

### 3.1. Thermogravimetric Analysis

**Figure 4:**
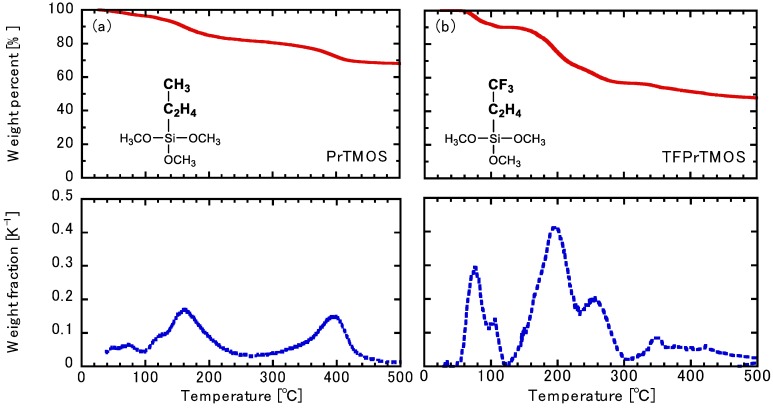
Thermogravimetric analysis of hydrolyzed silica powders (**a**) PrTMOS and (**b**) TFPrTMOS. (Note: Top graphs = the weight percent; bottom graphs = the dependence of weight fraction on temperature).

Figure 4 shows the decomposition trends of the organic groups within the PrTMOS and TFPrTMOS powders, investigated using TG analysis in an O_3_ atmosphere. The two topmost figures show the weight changes as a function of temperature, whereas the bottom two figures show the weight change differentials. Here, the weight change of PrTMOS was shown at both 150 °C and 400 °C, whereas those of TFPrTMOS were shown from 150 °C to 270 °C. Unfortunately, SiO_2_ did not decompose at the expected 500 °C temperature, where the difference in weight change over 500 °C was found to be small in both hydrolyzed silica powders. This means almost all the organic functional groups in the silica hydrolyzed powder decomposed completely, leaving only silica remaining at 500 °C. The organic groups CF_3_–C_2_H_4_ and C_3_H_7_ would, therefore, be attributed to the decomposition of PrTMOS and TFPrTMOS. The changes in weight for PrTMOS and TFPrTMOS were found to be 28.0% and 19.7%, respectively. If the hydrolysis of the silica precursors were to proceed completely, the atomic ratios of the Si and organic groups would be 1. Thus, the calculated weight changes to 45.2% and 65.1% for both PrTMOS and TFPrTMOS, respectively. Nevertheless, the weight changes shown in Figure 4 were found to be smaller than those calculated. This should not alter nor affect the decomposition temperature with this perceived difference in total decomposition amount.

The TFPrTMOS powder decomposition did, however, show more sharp peaks compared with the decomposition of the PrTMOS powders in Figure 4. We infer CF_3_–C_2_H_4_ to be easily decomposable compared with C_3_H_7_. We found the decomposition of CF_3_–C_2_H_4_ to occur around 120–230 °C and 230–300 °C, whereas the decomposition of C_3_H_7_ was found to start at 135–270 °C and finish around 270–460 °C. We ascertain the fluorocarbon group (–CF_3_) of CF_3_–C_2_H_4_ decomposed around 120–230 °C. This equates to a total weight change percent of 22.8% and 10.1% around 120–230 °C and 230–300 °C, respectively. The molecular weight ratio of –CF_3_ and C_2_H_4_ was found to be 2.5, which is similar to a ratio of 22.8% and 10.1% (=2.3). Thus, it was concluded that CF_3_ decomposed around 120–230 °C and C_2_H_4_ decomposed around 230–300 °C.

### 3.2. Gas Permeation Tests using TFPrTMOS-Derived Membranes

**Figure 5:**
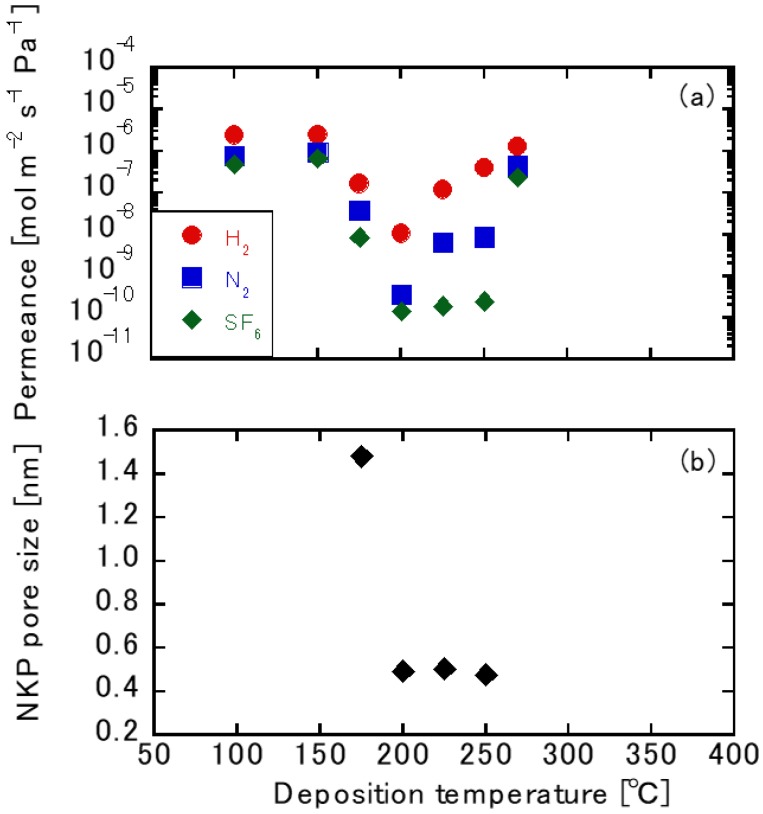
(**a**) The single gas permeation dependence on deposition temperature and (**b**) the normalized Knudsen-based permeance (NKP) pore size dependence on deposition temperature.

Figure 5a shows the single gas permeation tests using the TFPrTMOS-derived membranes deposited between 100 °C and 270 °C. Here, the pore sizes were evaluated using the normalized Knudsen-based permeance method [25]. We found the gas permeations through the membranes manipulated at 100 °C and 150 °C had a higher gas permeation performance compared with the γ-alumina substrate. This indicates that the reaction temperatures were set too low for silica deposition. On the other hand, the membranes deposited between 175 °C and 250 °C saw the H_2_ permeances reduce compared with the γ-alumina substrate, indicating that the TFPrTMOS decomposed successfully to form the membranes. The highest H_2_/SF_6_ permeance ratio among the seven membranes studied was 1645 from a membrane deposited at 250 °C. For H_2_, N_2_, and SF_6_, the permeances were 4.01 × 10^−7^ mol m^−2^ s^−1^ Pa^−1^, 8.41 × 10^−9^ mol m^−2^ s^−1^ Pa^−1^, and 2.44 × 10^−10^ mol m^−2^ s^−1^ Pa^−1^, respectively. The H_2_ permeance for the membrane deposited at 270 °C was also found to be high at around 1.28 × 10^−6^ mol m^−2^ s^−1^ Pa^−1^, which was slightly lower than that of the γ-alumina substrate. The deposition temperature for the TFPrTMOS was between 175 °C and 270 °C.

Figure 5b shows the relationship between the pore size and deposition temperature for PrTOMS and TFPrTMOS. The pore size was evaluated using the permeance ratios of N_2_ and SF_6_ over that of H_2_. Here, the pore size was found to differ only between 175 °C and 250 °C, as shown in Figure 5b. The smallest pore size attained was around 0.48 nm from the membrane deposited between 200 °C and 250 °C. This could be due to the balance between deposition rate and decomposition rate as the deposition temperature changed. As discussed previously, the deposition rate was expected to be higher for the higher deposition temperature. However, C_2_H_4_ and CF_3_–C_2_H_4_ each decomposed at around 250 °C. The pores also hardly formed below 200 °C, with a temperature over 250 °C found to be needed to obtain larger pores. This trend was also discussed for the deposition of PrTMOS in other research work [19].

### 3.3. Liquid Permeation Tests using the TFPrTMOS-Derived Membranes

#### 3.3.1. NaCl Permeation

**Figure 6:**
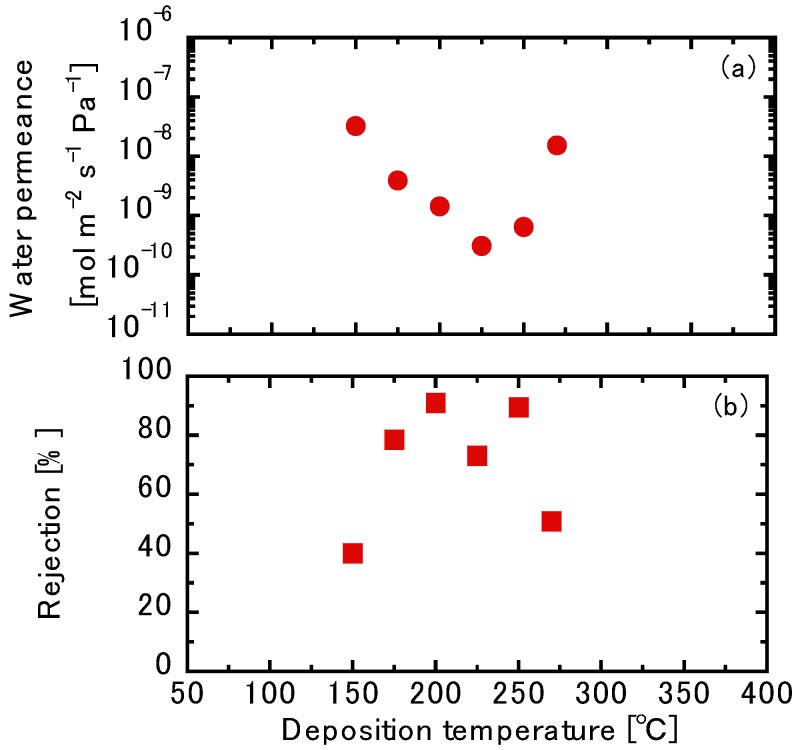
(**a**) The water permeance and (**b**) the NaCl rejection of TFPrTMOS-derived membranes.

Figure 6 shows the water permeations as well as the NaCl rejections using the TFPrTMOS-derived membranes deposited between 150 °C and 270 °C. Here, the water showed a minimum permeation at 3.06 × 10^−10^ mol m^−2^ s^−1^ Pa^−1^ through the membrane deposited at 225 °C. The other water permeances through these membranes also showed similar trends, particularly for those of SF_6_, as shown in Figure 5. Detailed discussions on this will be described later. The highest NaCl rejection was found to be 91.0%, which was shown through the membrane deposited at 200 °C. These high NaCl rejections could be explained roughly by the recognized low water permeations.

As mentioned previously, the pore sizes of the membranes deposited between 200 °C and 250 °C were around 0.48–0.50 nm, whereas the hydrated diameters of Na^+^ and Cl^−^ were determined to be 0.72 and 0.66 nm, respectively. The high rejections through the membranes in this case can be explained by molecular sieving. Nevertheless, the NaCl with a pore size of 1.48 nm had a rejection of 78.4% through the membrane deposited at 175 °C. Unfortunately, it is difficult to discuss this effect by molecular sieving only.

#### 3.3.2. Relationship between the Gas and Liquid Permeation Performance

**Figure 7:**
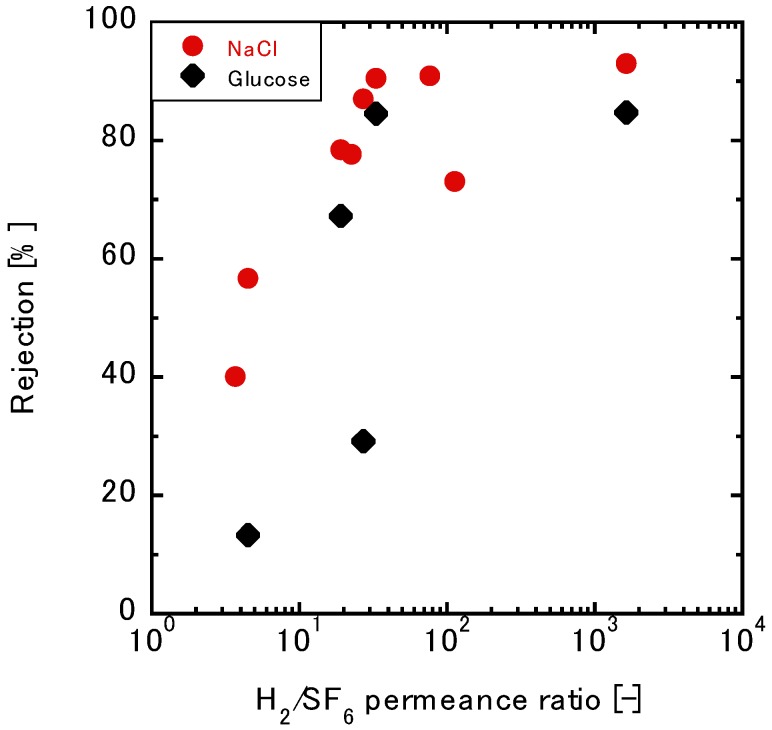
The relationship between the gas permeance ratio and the rejection of glucose through the TFPrTMOS-derived membrane.

Figure 7 shows the relationship between the NaCl, glucose rejections, and the H_2_/SF_6_ permeance ratios. It is well known that glucose is a neutral molecule without charge effects in the presence of water. Here, we see the glucose rejection increase with an increase in the H_2_/SF_6_ permeance ratios up until a H_2_/SF_6_ ratio of 30, which indicates that both SF_6_ and glucose permeations are based on the molecular sieving mechanism. The maximum rejection was 84.6%. The molecular weight of glucose in this case was more than twice that of NaCl. Notwithstanding, the maximum NaCl rejection was 91.0%, which was higher than that of glucose. Thus, charge exclusion effects should be considered during permeations. This will be discussed using multivalent ions in the following section.

#### 3.3.3. Permeation of the Multivalent Ions

**Figure 8:**
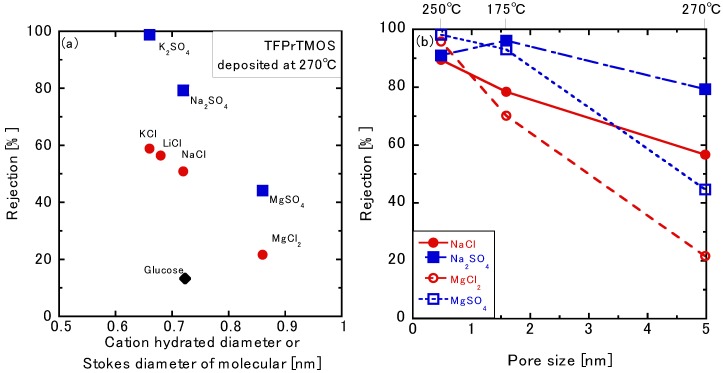
(**a**) Relation between ion rejection and hydrated ion diameter (or Stokes diameter of glucose) for TFPrTMOS-derived membranes deposited at 270 °C. (**b**) The relationship between the pore size and ion rejections of the TFPrTMOS-derived membrane deposited at 175 °C, 250 °C, and 270 °C.

**Figure 9:**
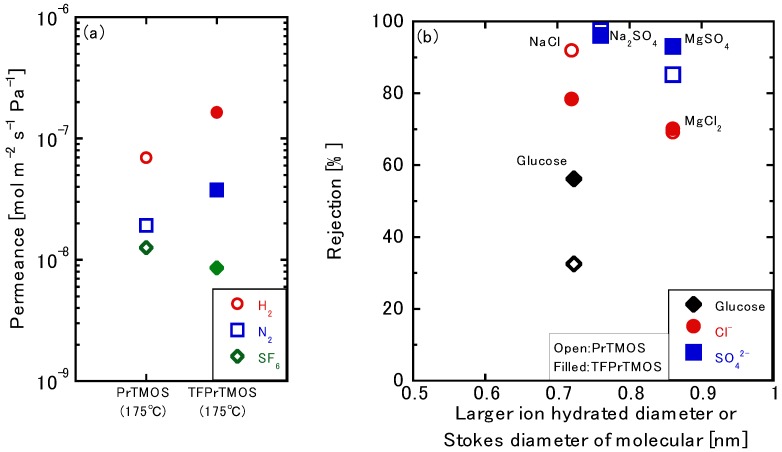
(**a**) The gas permeance and (**b**) the ion rejection through PrTMOS-derived and TFPrTMOS-derived membrane deposited at 175 °C.

Figure 8 shows the liquid permeation tests using mono and multivalent ion solutions of the TFPrTMOS-derived membranes deposited at 175 °C, 250 °C, and 270 °C. As shown in Figure 6, high NaCl rejection of 89.5% was obtained for the membrane deposited at 250 °C. The rejections for Na_2_SO_4_, MgCl_2_, and MgSO_4_ were 91.0%, 95.8%, and 98.2%, respectively. Systematically, the order of these rejections was MgSO_4_ > MgCl_2_ > Na_2_SO_4_ > NaCl, as shown in Table 3. Here, Mg^2+^ rejections were the highest among the cations, whereas the SO_4_^2−^ rejections were found the highest among the anions. The rejections through the membrane deposited at 175 °C for NaCl, Na_2_SO_4_, MgCl_2_, and MgSO_4_ were 78.0%, 96.2%, 70.1%, and 93.0%, respectively. The rejection order was Na_2_SO_4_ > MgSO_4_ > NaCl > MgCl_2_. The average pore size was also found to be 1.48 nm, which is larger than that of the membrane deposited at 250 °C. Negatively charged polymer commercial nanofiltration (molecular weight cutoff: 1000–2000) also showed the same ordering at Na_2_SO_4_ > MgSO_4_ > NaCl > MgCl_2_, which is the same order as the membrane deposited at 175 °C. Through the negatively charged membranes, SO_4_^2−^ rejections were the highest among the anions, whereas the Mg^2+^ rejections were found the lowest among the cations [26]. In addition, the pore size 1.48 nm was much larger than the hydrated ion size. Thus, we attribute the permeation mechanism for the membrane deposited at 175 °C as charge exclusion. Conversely, the order was different with the 89.5% rejection found for the membrane deposited at 250 °C. Therefore, molecular sieving would also be considered as the permeation mechanism due to the smaller pore sizes achieved with the membrane deposited at 250 °C. That was confirmed by the higher rejections found for MgSO_4_ and MgCl_2_ that included the ion with the largest hydrated diameter (i.e., Mg^2+^). The rejections for the membrane deposited at 270 °C for NaCl, Na_2_SO_4_, MgCl_2_, and MgSO_4_ were 56.7%, 79.3%, 21.6%, and 44.6%, respectively. The order of this rejection was Na_2_SO_4_ > NaCl > MgSO_4_ > MgCl_2_. The pore size found for the membrane deposited at 270 °C was also the largest among the three membranes, as shown in Figure 9 from the gas and liquid permeation tests. As the organic groups in the silica separation layer decomposed, these were likely replaced by hydroxyl groups. The hydroxyl groups could also easily dissociate during testing to produce a negatively charged surface. Such negative surface charge effects for the membrane deposited at 270 °C were thought to be strong, particularly as the Mg^2+^ rejections were lower than the Na^+^ rejections.

#### 3.3.4. Effects of the Silica Precursors

Both PrTMOS- and TFPrTMOS-derived membranes deposited at 175 °C were compared through ion permeation. The NaCl, Na_2_SO_4_, MgCl_2_, and MgSO_4_ rejections of the PrTMOS-derived membranes were 92.0%, 97.4%, 69.2%, and 85.4%, respectively. The glucose rejection was only 35.9%, whereas that of the TFPrTMOS-derived membrane was 67.2%. The order of the rejection was found to be Na_2_SO_4_ > NaCl > MgSO_4_ > MgCl_2_, which was different from that of the TFPrTMOS-derived membrane, as shown previously. The order was also the same as the membrane deposited at 270 °C. Figure 9a shows the single gas permeations of the PrTMOS- and TFPrTMOS-derived membranes. The H_2_ permeance of the PrTMOS-derived membrane was found to be smaller than that of the TFPrTMOS-derived membrane. However, the permeation ratios of H_2_/N_2_ and H_2_/SF_6_ were shown to be 3.6 and 5.5, indicating that the permeation may be based on Knudsen diffusion. This may explain why the pore size was similar to that of the TFPrTMOS-derived membrane deposited at 270 °C. At the same deposition temperature, TFPrTMOS was found to generate much smaller pores than PrTMOS. This means that TFPrTMOS deposits more easily than PrTMOS because of its higher reactivity. The decomposition temperature of C_3_H_7_ within PrTMOS was 100–200 °C, as shown in Figure 4. Therefore, C_3_H_7_ should also decompose at 175 °C to form hydroxyl groups inside the separation layer. The permeation properties of the PrTMOS-derived membrane were also similar to those of the TFPrTMOS-derived membrane deposited at 270 °C. We found the pore size and organic group decomposition to be the most important factors for ion permeation.

## 4. Conclusions

In this study, TFPrTMOS membranes were deposited successfully at a temperature higher than 175 °C. The TFPrTMOS membrane that had the highest H_2_/SF_6_ permeance ratio (H_2_/SF_6_ = 1645) was found to be at 250 °C. Here, NaCl rejections were shown to improve around 175–250 °C. Ion rejections were controlled by the decomposition temperature due to the decomposition of the organic groups in the silica precursors. The NaCl had a rejection of 91.0%, which was obtained through the membrane deposited at 200 °C. We infer the TFPrTMOS membranes to be negatively charged. The ion permeation mechanism was found to change due to charge effects with membranes having a pore size of 1.48 nm or above. The TFPrTMOS membrane showed higher glucose rejection at 67.2%, in comparison with the PrTMOS membrane. The pore size and the decomposition of the organic groups were found to be important for ion permeation.

## Figures and Tables

**Table 1:** Solutes utilized for the liquid permeation test.

Solute	Manufacturer and Purity	MW [g mol^−1^]
D-Glucose	FUJIFILM Wako Pure Chemicals Co., Ltd.	180.16
NaCl	Sigma-Aldrich, 99.98% trace metals basis	74.55
Na_2_SO_4_	Merck, ≥99.0%	142.04
MgCl_2_	Merck, ≥99.0%	95.21
MgSO_4_	Sigma-Aldrich, ACS reagent, ≥98%	120.37

**Table 2:** The hydrated ion diameters of Na^+^, Mg^2+^, Cl^-^ and SO_4_^2-^ [24].

Cation	Hydrated Diameter [nm]	Anion	Hydrated Diameter [nm]
Na^+^	0.72	Cl^−^	0.66
Mg^2+^	0.86	SO_4_^2−^	0.76

**Table 3:** The order of ion rejection, glucose rejection, and pore size in silica and polymeric membranes.

Membranes	Deposition Temperature [°C]	The Order of ion Rejections	Glucose Rejection [%]	Pore Size [nm]
TFPrTMOS	175	Na_2_SO_4_ > MgSO_4_ > NaCl > MgCl_2_	67.2	1.48
	250	MgSO_4_ > MgCl_2_ > Na_2_SO_4_ > NaCl	84.7	0.48
	270	Na_2_SO_4_ > NaCl > MgSO_4_ > MgCl_2_	13.3	1.48<
PrTMOS	175	Na_2_SO_4_ > NaCl > MgSO_4_ > MgCl_2_	35.9	1.48<
Polymeric membrane [26]	-	Na_2_SO_4_ > MgSO_4_ > NaCl > MgCl_2_	-	-

## References

[1] Tsuru T. (2001). Inorganic porous membranes for liquid phase separation. Sep. Purif. Technol..

[2] Hubadillah S.K., Othman M.H.D., Matsuura T., Ismail A.F., Rahman M.A., Harun Z., Jaafar J., Nomura M. (2018). Fabrications and applications of low cost ceramic membrane from kaolin: A comprehensive review. Ceram. Int..

[3] Yuan B., Li P., Wang P., Yang H., Sun H., Li P., Sun H., Niu Q.J. (2019). Novel aliphatic polyamide membrane with high mono-/ divalent ion selectivity, excellent Ca^2+^, Mg^2+^ rejection, and improved antifouling properties. Sep. Purif. Technol..

[4] Sugiyama Y., Ikarugi S., Oura K., Ikeda A., Matsuyama E., Ono R., Nomura M., Tawarayama H., Saito T., Kuwahara K. (2015). MFI Zeolite Membranes Prepared on Novel Silica Substrates. J. Chem. Eng. Jpn..

[5] Sakai M., Sasaki Y., Tomono T., Seshimo M., Matsukata M. (2019). Olefin Selective Ag-Exchanged X-Type Zeolite Membrane for Propylene/Propane and Ethylene/Ethane Separation. ACS Appl. Mater. Interfaces.

[6] Zhou M., Chen X., Kita H. (2010). Inexpensive synthesis of silicalite-1 membranes with high pervaporation performance. Chem. Lett..

[7] Kato H., Lundin B.S., Ahn S., Takagaki A., Kikuchi R., Oyama T. (2019). Gas Separation Silica Membranes Prepared by Chemical Vapor Deposition of Methyl-Substituted Silanes. Membranes.

[8] Nishida R., Tago T., Saitoh T., Seshimo M., Nalao S. (2019). Development of CVD Silica Membranes Having High Hydrogen Permeance and Steam Durability and a Membrane Reactor for a Water Gas Shift Reaction. Membranes.

[9] Zhu B., Morris G., Moon I., Gray S., Duke M. (2018). Diffusion behavior of multivalent ions at low pH through a MFI-type zeolite membrane. Desalination.

[10] Elma M., Yacou C., Costa D.C.J., Wang K.D. (2013). Performance and Long Term Stability of Mesoporous Silica Membranes for Desalination. Membranes.

[11] Xu R., Kanezashi M., Yoshioka T., Okuda T., Ohshita J., Tsuru T. (2013). Tailoring the affinity of organosilica membranes by introducing polarizable ethenylene bridges and aqueous ozone modification. ACS Appl. Mater. Interfaces.

[12] Nomura M., Ono K., Gopalakrishnan S., Sugawara T., Nakao S. (2005). Preparation of a stable silica membrane by a counter diffusion chemical vapor deposition method. J. Membr. Sci..

[13] Myagmarjav O., Ikeda A., Tanaka N., Kubo S., Nomura M. (2017). Preparation of an H_2_-permselective silica membrane for the separation of H_2_ from the hydrogen iodide decomposition reaction in the iodine-sulfur process. Int. J. Hydrogen Energy.

[14] Ishii K., Shibata A., Takeuchi T., Yoshiura J., Urabe T., Kameda Y., Nomura M. (2019). Development of silica membranes to improve dehydration reactions. J. Jpn. Petrol. Inst..

[15] Myagmarjav O., Tanaka N., Nomura M., Kubo S. (2019). Module design of silica membrane reactor for hydrogen production via thermochemical IS process. Int. J. Hydrogen Energy.

[16] Myagmarjav O., Tanaka N., Nomura M., Kubo S. (2017). Hydrogen production tests by hydrogen iodide decomposition membrane reactor equipped with silica-based ceramics membrane. Int. J. Hydrogen Energy.

[17] Ikeda A., Nomura M. (2016). Preparation of amorphous silica based membranes for separation of hydrocarbons. J. Jpn. Petrol. Inst..

[18] Ikeda A., Ono R., Nomura M. (2015). High hydrogen permeance silica membranes prepared by a chemical vapor deposition method. J. Membr. Sep. Tech..

[19] Matsuyama E., Ikeda A., Komatsuzaki M., Sasaki M., Nomura M. (2014). High temperature propylene/propane separation through silica hybrid membranes. Sep. Purif. Technol..

[20] Nomura M., Momma K., Negishi Y., Matsuyama E., Kimura S. (2010). Preparation of silica hybrid membranes for high temperature gas separation. Desalin. Water Treat..

[21] Nomura M., Nagayo T., Monma K. (2007). Pore size control of a molecular sieve silica membrane prepared by a counter diffusion CVD method. J. Chem. Eng. Jpn..

[22] Ikeda A., Matsuyama E., Komatsuzaki M., Sasaki M., Nomura M. (2014). Development of inorganic silica reverse osmosis membranes by using a counter-diffusion chemical vapor deposition method. J. Chem. Eng. Jpn..

[23] Ishii K., Ikeda A., Takeuchi T., Yoshiura J., Nomura M. (2019). Silica-based RO membranes for separation of acidic solution. Membranes.

[24] Nightingale E.R. (1959). Phenomenological theory of ion solvation. Effective radii of hydrated ions. J. Phys. Chem..

[25] Lee H.R., Kanezashi M., Shimomura Y., Yoshioka T., Tsuru T. (2011). Evaluation and fabricatioin of pore-size-tuned silica membranes with tetraethoxydimethyl disiloxane for gas separation. AlChE J..

[26] Tsuru T. (1995). Ion Separation by Charged Membranes in Reverse Osmosis. Membrane.

